# *myo*‐Inositol's role in laying hens’ active gastrointestinal microbiota: a strain comparison

**DOI:** 10.1002/jsfa.70984

**Published:** 2026-08-15

**Authors:** Naomi Sarpong, Christoph Roth, Markus Rodehutscord, Jana Seifert, Amélia Camarinha‐Silva

**Affiliations:** ^1^ Institute of Animal Science University of Hohenheim Stuttgart Germany; ^2^ HoLMiR – Hohenheim Center for Livestock Microbiome Research University of Hohenheim Stuttgart Germany

**Keywords:** active gastrointestinal microbiota, laying hens, *myo*‐inositol, 16S rRNA sequencing

## Abstract

**BACKGROUND:**

*myo*‐Inositol (MI) is a cyclitol involved in cellular metabolism and phytate degradation, yet its influence on the gastrointestinal tract (GIT) microbiota of laying hens remains poorly understood. This study investigated the effects of graded dietary MI supplementation on the active GIT microbial communities of two commercial laying hen strains.

**RESULTS:**

Lohmann Brown Classic (LB) and Lohmann LSL‐Classic (LSL) hens were fed diets supplemented with 0, 1, 2, or 3 g MI kg^−1^ feed. Active microbiota were assessed by 16S rRNA gene amplicon sequencing (cDNA‐based) of 623 mucosa and digesta samples from the crop, duodenum, ileum, and ceca. Microbial community composition was primarily shaped by GIT section (*R* ^2^ = 0.43), followed by host strain (*R* ^2^ = 0.03) and sample type (*R* ^2^ = 0.02). MI supplementation explained a smaller proportion of variance but significantly influenced specific taxa, particularly in the crop and ceca. Overall, 66 amplicon sequence variants, including members of *Limosilactobacillus*, *Bacteroides*, and *Roseburia*, differed across MI concentrations. Strain‐dependent responses were observed, with LB hens showing greater separation between mucosa and digesta communities, whereas LSL hens exhibited a higher number of MI‐responsive amplicon sequence variants in the duodenal and ileal digesta. Correlation analyses revealed associations between *Ligilactobacillus* and egg number and yolk MI concentration, and between *Alistipes* and egg weight.

**CONCLUSION:**

Dietary MI supplementation induces localized and strain‐dependent alterations in the active GIT microbiota of laying hens. Although these effects were modest compared with host genotype and GIT section, the findings provide first evidence for MI–microbiota interactions with potential relevance to egg production traits. © 2026 The Author(s). *Journal of the Science of Food and Agriculture* published by John Wiley & Sons Ltd on behalf of Society of Chemical Industry.

**ABBREVIATIONS**
ADFI_Whole_
: average daily feed intake (whole period)
ASV: amplicon sequence variant
BH: Benjamini–Hochberg
BW_Slaughter_
: body weight at slaughter
GIT: gastrointestinal tract
LB: Lohmann Brown Classic
LSL: Lohmann LSL‐Classic
MI: *myo*‐inositol
MI0: diet without *myo*‐inositol supplementation
MI1: diet supplemented with 1 g of *myo*‐inositol kg^−1^ feed
MI2: diet supplemented with 2 g of *myo*‐inositol kg^−1^ feed
MI3: diet supplemented with 3 g of *myo*‐inositol kg^−1^ feed
NMDS: non‐metric multidimensional scaling
PERMANOVA: permutational multivariate analysis of variance
rCLR: robust centered log‐ratio

## INTRODUCTION

*myo*‐Inositol (MI), a cyclitol occurring in free form or bound in phospholipids and inositol phosphates in animals and plants, contributes to cellular signaling, osmoregulation, and lipid metabolism.^1^ The intestinal capacity to release MI by complete degradation of phytate depends on both host mucosal enzymes and microbial enzyme activity.^2^ The dietary supply of free MI supplementation leads to higher MI concentrations in the intestinal digesta of laying hens, but its impact on the gut microbiota remains unclear.^3^ Certain intestinal bacteria possess the genetic machinery to utilize MI as a carbon source, potentially conferring a competitive advantage in the presence of high luminal MI concentrations.^4^, ^5^

The gastrointestinal tract (GIT) of laying hens has diverse morphological and physiological characteristics,^6^ resulting in complex microbiota communities along the different GIT sections.^7^ Gut microbiota significantly influences the physiological status, productivity, and overall performance of chickens, with greater taxonomic diversity and balanced community structures often associated with improved digestive function, nutrient digestibility, and disease resistance.^8^ Previous studies in laying hens have shown that diet and hen genetic strain interact to influence the microbiota composition. Variation in calcium and phosphorus supply affected microbial communities differently in Lohmann Brown Classic (LB) and Lohmann LSL‐Classic (LSL) strains,^9^ and strain × diet interactions were also observed in cecal microbiota when comparing high‐ and low‐feed‐efficiency hens under control or high‐fiber diets.^10^ Such responses suggest that microbial taxa, including *Alistipes* and *Anaerosporobacter*, may contribute to nutrient utilization and host metabolism in a strain‐dependent manner.

Associations between GIT microbiota composition and laying performance have been reported, with high egg production linked to greater abundances of Bacillota, Pseudomonadota and *Lactobacillus*, whereas lower production was correlated with higher alpha diversity and more complex community architectures.^9^, ^11^, ^12^ Interestingly, higher *Lactobacillus* abundance in LB hens was associated with lower egg weights.^9^ Similarly, probiotic supplementation (mixture of *Bacillus subtilis*, lactic acid bacteria and *Saccharomyces*) has been shown to enhance performance, egg quality, ovarian development, and immune function throughout the laying cycle,^12^ supporting the association between specific species and egg performance.

The present study characterized the active microbial communities in mucosa and digesta from the crop, duodenum, ileum, and ceca of LB and LSL hens fed graded MI‐supplemented diets. We aimed to determine whether dietary MI supplementation alters the active GIT microbiota, and whether responses differ between hen strains, GIT sections, and sample types. Because dietary MI may directly influence microbial metabolism, we used cDNA‐based 16S rRNA sequencing to target the metabolically active microbiota rather than the total microbial community.

## MATERIAL AND METHODS

### Sampling, DNA extraction, and sample preparation for target amplicon sequencing

This study was conducted as part of the interdisciplinary Research Unit P‐Fowl (FOR2601) (https://p-fowl.uni-hohenheim.de/). The animal trial was approved by the Regierungspräsidium Tübingen, Germany (Project No. HOH67‐21TE) and conducted at the Agricultural Experiment Station of the University of Hohenheim in accordance with German animal welfare legislation. A detailed description of the experimental setup is provided by Sommerfeld *et al*.^3^

Briefly, 240 female chicks of each strain (LB and LSL) were raised under standardized conditions (floor pens with deep litter bedding). At 26 weeks of age, hens were moved to individual metabolic units (1 m × 1 m × 1 m) arranged in a randomized complete block design (10 replicate hens per strain and dietary treatment). Each individually housed hen represented one experimental unit. The corn–soybean meal‐based diets were formulated according to the recommendations of the Gesellschaft für Ernährungsphysiologie,^13^ except for non‐phytate phosphorus, which was provided at 2.2 g kg^−1^, as suggested by Rodehutscord *et al*.^14^ Diets were either unsupplemented (MI0) or supplemented with 1 (MI1), 2 (MI2), or 3 g MI kg^−1^ feed (MI3), simulating half to full release of MI from InsP_6_ or a therapeutic human dose (Tables 1 and 2). Diets were fed ad libitum for 4 weeks (26–30 weeks of age). During this 27‐day period, eggs were collected daily for counting (egg number) and average weight determination (mean egg weight). One egg per hen laid in the week before slaughter was stored for measurement of the MI concentration in the albumen (MI conc. albumen) and yolk (MI conc. yolk). Average daily feed intake (ADFI_Whole_) was calculated over the entire time spent in the metabolic units. For microbiota analysis, one hen from each replicate was sampled at the end of the feeding trial (10 hens per strain × dietary treatment; *n* = 80). On each slaughter day, hens (*n* = 20) were fasted for 1 h and then allowed ad libitum access to feed for 1 h to standardize gut fill. Body weight at slaughter day (BW_Slaughter_) was recorded. Birds were euthanized by decapitation after stunning with a gas mixture (35% CO_2_, 35% N_2_, and 30% O_2_). The average egg weight in the oviduct of each hen was measured (egg weight oviduct). From each hen, paired digesta and mucosa samples were collected from the crop, duodenum, ileum, and ceca, resulting in eight samples per bird (80 hens × 8 samples) for active microbiota analysis. All tissues were opened under sterile conditions. Digesta was collected using a sterile spoon, and mucosa was scraped using a sterile glass slide. Samples were immediately stored in RNAlater at −80 °C until further processing. Sommerfeld *et al*.^3^ collected duodenal mucosa for phosphatase activity determination, and ileal digesta and duodenal and jejunal digesta together (duodenum+jejunum) for MI concentration measurements.

**Table 1:** Ingredient composition of the dietary treatments^a^

Ingredient (g kg^−1^)	MI0	MI1	MI2	MI3
Corn	591	590	589	588
Extracted soybean meal	260	260	260	260
Alfalfa meal	30	30	30	30
Soybean oil	20	20	20	20
dl‐Methionine	3.5	3.5	3.5	3.5
Monocalcium phosphate	4.7	4.7	4.7	4.7
Limestone, fine	23	23	23	23
Limestone, coarse	54.4	54.4	54.4	54.4
Sodium chloride	3	3	3	3
Choline chloride	1	1	1	1
Sodium bicarbonate	1.9	1.9	1.9	1.9
Vitamin mix	2	2	2	2
Mineral mix	0.5	0.5	0.5	0.5
*myo*‐Inositol	0	1	2	3
TiO_2_	5	5	5	5

^a^ Detailed descriptions of ingredients and analytical composition of the diets are given in the companion article.^3^

**Table 2:** Chemical composition of the dietary treatments^a^

Ingredients (g kg^−1^)	MI0	MI1	MI2	MI3
Corn	591	590	589	588
Extracted soybean meal	260	260	260	260
Alfalfa meal	30	30	30	30
Soybean oil	20	20	20	20
dl‐Methionine	3.5	3.5	3.5	3.5
Monocalcium phosphate	4.7	4.7	4.7	4.7
Limestone, fine	23	23	23	23
Limestone, coarse	54.4	54.4	54.4	54.4
Sodium chloride	3	3	3	3
Choline chloride	1	1	1	1
Sodium bicarbonate	1.9	1.9	1.9	1.9
Vitamin mix^b^	2	2	2	2
Mineral mix^c^	0.5	0.5	0.5	0.5
*myo*‐Inositol	0	1	2	3
TiO_2_	5	5	5	5
Calculated (g kg^−1^)				
*myo*‐Inositol	0.4	1.4	2.4	3.4
P	4	4	4	4
*Non* phytate‐P	2.2	2.2	2.2	2.2
Ca	35	35	35	35
CP	168	168	168	168
ME (MJ kg^−1^)	11.6	11.6	11.6	11.6
Analyzed (g kg^−1^ DM)				
*myo*‐Inositol	0.37	1.51	2.76	3.72
P	4.4	4.5	4.4	4.5
Ca	34.6	36.3	33.3	33.3

^a^ Detailed descriptions of ingredients and analytical composition of the diets are given in the companion article.^3^

^b^ Vitamin premix provided per kg of complete diet: vitamin A, 10000 IU; vitamin D₃, 3000 IU; vitamin E, 30 mg; vitamin K_3_, 2.4 mg; biotin, 100 μg; folic acid, 1 mg; vitamin B_1_, 3 mg; vitamin B_2_, 6 mg; vitamin B_6_, 6 mg; vitamin B_12_, 30 μg; nicotinamide, 50 mg; calcium d‐pantothenate, 14 mg.

^c^ Trace element premix provided per kg of complete diet: manganese, 80 mg (from manganese oxide); zinc, 60 mg (from zinc sulfate monohydrate); iron, 25 mg (from ferrous sulfate monohydrate); copper, 7.5 mg (from cupric sulfate pentahydrate); iodine, 0.6 mg (from calcium iodate); selenium, 0.2 mg (from sodium selenite).

RNA was extracted from a total of 640 samples obtained from 80 hens (10 hens per strain × dietary treatment) using Trizol reagent (Invitrogen Inc., Carlsbad, CA, USA), following the manufacturer's instructions, with a preliminary bead‐beating step (30 s, 5.5 m s^−1^) using a FastPrep instrument (MP Biomedicals, Eschwege, Germany). RNA concentration was quantified using a Nanodrop spectrophotometer (Thermo Fisher Scientific, Waltham, MA, USA) and stored at −80 °C until further analysis. Samples were treated with DNase (Invitrogen), to remove genomic DNA contamination, and cDNA was synthesized using the SuperScript III First‐Strand Synthesis System for RT‐PCR (Invitrogen). Target amplicon sequencing of the V1–V2 region of the 16S rRNA gene was performed following Borda‐Molina *et al*.^15^ Library preparation was conducted using the PrimeSTAR HS DNA Polymerase kit (TaKaRa, Beijing, China). The first two PCR reactions were prepared in a total volume of 25 μL, containing 1 μL cDNA template, 0.2 μmol L^−1^ of each primer, and 0.5 U PrimeSTAR HS DNA polymerase. The third PCR was set up in a 50 μL volume. Thermocycling conditions included initial denaturation at 95 °C for 3 min, followed by either 10 cycles (first and second PCR) or 20 cycles (third PCR) of denaturation at 98 °C for 10 s, annealing at 55 °C for 10 s, and extension at 72 °C for 45 s, with a final extension at 72 °C for 2 min. Amplicons were verified by agarose gel electrophoresis, purified, and normalized using the SequalPrep Normalization Kit (Invitrogen). Sequencing was carried out using 250 bp paired‐end reads on a NovaSeq 6000 platform (Illumina, San Diego, CA, USA. A cDNA‐based 16S rRNA amplicon sequencing approach was chosen to characterize the metabolically active microbial community, which was the primary focus of this study. Ribosomal RNA is generally more abundant in metabolically active cells and therefore provides complementary information to DNA‐based community profiling, which also includes dormant and dead microorganisms. This approach was considered particularly suitable for investigating microbial responses to dietary MI supplementation.

### Bioinformatical and statistical analysis

Target amplicon sequencing data were analyzed with Qiime2, after demultiplexing Fastq files with Sabre (v.2023.9).^16^ Reads were trimmed using the q2‐cutadapt plugin.^17^ The q2‐dada2 plugin identified amplicon sequence variants (ASVs) through quality filtering, error correction, dereplication, and merging of reads.^18^ ASVs with sequence length lower than 100 bp and/or that occurred in fewer than ten samples were deleted. Sklearn‐based classifiers^19^ were generated with RESCRIPt^20^ using the Silva SSU‐rRNA database (v.138.1, 16S 99%).^21^ These were used for the taxonomic assignment of ASVs using VSEARCH.^22^ Samples containing ASVs with a sequence length of less than 200 bp were deleted. Reads from organelles and unassigned sequences were excluded from the analysis.

The individual hen was considered the experimental unit of the feeding trial. From each hen, paired mucosa and digesta samples were collected from four GIT sections, resulting in repeated observations within individual hens. For the overall community analysis, a permutational multivariate analysis of variance (PERMANOVA) was performed. Hen identity was used as the permutation stratum to account for the repeated sampling structure. ASV‐level analyses were performed separately for each strain, GIT section, and sample type, such that each statistical model included only one observation per hen.

The statistical analysis of the target amplicon sequencing data was done using R version 4.4.0 (2024‐04‐24).^23^ Shannon diversity index was calculated with ‘vegan’ to assess the alpha diversity in the samples.^24^ The total number of reads was standardized per sample and the Bray–Curtis dissimilarity coefficient was used to create a sample dissimilarity matrix. The influence of the factors ‘strain’, ‘GIT section’, and ‘sample type’ (mucosa or digesta) on the microbial community of the entire data set was evaluated by calculating PERMANOVA using the ‘adonis’ function of the package ‘vegan’ at the ASV level.^24^ Hen identity was included as the permutation stratum to account for repeated observations within individual hens. The entire data set was subdivided by ‘GIT section’ and either by ‘strain’ or ‘sample type’. Non‐metric multidimensional scaling (NMDS) was used for the visualization of sample ordination and clustering within both subdivisions, and the results were plotted using the ‘ggplot2’ package.^25^ PERMANOVA was calculated with the aforementioned approach to explore the influence of factors ‘diet’ and ‘sample type’ within the GIT section of each strain, and the influence of factors ‘diet’ and ‘strain’ within the GIT section of each sample type.

To identify taxa responding to MI concentrations, the data set was stratified by ‘strain’, ‘GIT section’, and ‘sample type’. Relative abundances within each subset were robust centered log‐ratio (rCLR) transformed to approximate normality and account for compositionality. Outliers were excluded using the IQR method.^26^ Cleaned data sets were analyzed with linear models including ‘diet’ (MI concentration) as the fixed factor, using the ‘lme4’ package.^27^ To reveal MI concentration differences, pairwise comparisons were performed using Tukey tests.^28^ Results were visualized using the package ‘ComplexHeatmap’.^29^ At the genus level, differences between ‘strains’ within the same MI concentration were tested using the Wilcoxon test with Benjamini–Hochberg (BH) adjustment for multiple comparisons.

To standardize across data sets, Z‐scores were calculated for the zootechnical parameters (BW_Slaughter_ and ADFI_Whole_), egg parameters (egg number, mean egg weight, egg weight oviduct, MI conc. albumen, MI conc. yolk), parameters of MI metabolism (phosphatase, ileal and duodenum+jejunum MI conc.), and target amplicon sequencing data at the genus level (average relative abundance higher than 1%, unclassified genera were excluded).^25^ As already mentioned, relative‐abundance data from the jejunum or the combined duodenum+jejunum digesta were not determined. Correlations with the MI concentrations in the duodenum+jejunum digesta determined by Sommerfeld *et al*.^3^ were performed using the microbial profiles from the duodenum digesta. The Spearman correlation coefficients were calculated between the *Z*‐scores within each ‘GIT section’. The visualization was done with the R package svglite.^30^ Only significant correlations (*P* < 0.05) were included. Wilcoxon test with BH procedures were used to compare the Shannon diversity index between ‘strains’. The Kruskal–Wallis test, followed by BH correction, was used to compare MI concentrations within each ‘GIT section’, ‘sample type’ and ‘strain’. Results were plotted using the ‘ggplot2’ package.^25^ All adjusted *P*‐values were indicated as *P*_adj_.

## RESULTS

### GIT section and hen strain drive the active microbiota structure

The microbiota data set comprised samples from 80 hens (10 hens per strain × dietary treatment). Paired digesta and mucosa samples were collected from four GIT sections, resulting in 640 planned samples. Following quality control, one sample was excluded, leaving 623 samples for downstream analyses. On average, 24 281 ± 533 (SEM) reads per sample were obtained, yielding a total of 1946 ASVs. Alpha diversity (Shannon diversity index) remained largely stable across dietary treatments, differing significantly only in duodenal mucosa under MI1, where LB showed lower values than LSL (1.9 *vs*. 3.1, *P*_adj_ < 0.05) (Supporting Information, Fig. S1A). No significant differences were detected between MI concentrations within one strain (*P*_adj_ > 0.05; Supporting Information, Fig. S1B). Based on Fig. S1B, the LB group showed lower mean microbial diversity in the duodenal and ileal mucosa, along with markedly higher inter‐individual variation. Although these differences did not reach statistical significance, the observed pattern suggests strain‐specific differences in microbial heterogeneity in the duodenum and ileum.

The PERMANOVA on the entire data set showed significant effects of ‘strain’, ‘GIT section’, and ‘sample type’, as well as their interactions (*P* < 0.05) (Supporting Information, Table S2). ‘GIT section’ explained the largest part of the variation (*R* ^2^ = 0.43), followed by ‘strain’ (*R* ^2^ = 0.03) and ‘sample type’ (*R* ^2^ = 0.02).

The NMDS ordination at the ASV level showed an uneven spatial distribution of the centroids corresponding to the different MI concentrations (Fig. 1). Strain‐specific clustering patterns emerged in the small intestine. The distances of the centroids for the MI concentrations were larger in the mucosa of the small intestine sections of the LB than LSL hens, indicating a more diverse active microbiota in the mucosa of the LB than LSL hens when supplemented with different levels of MI. Specifically, in LB hens, varying MI concentrations led to greater centroid dispersion in the small intestinal mucosa, suggesting a more dynamic microbial response to diet than the stable clustering observed in LSL. In contrast, the ceca samples grouped primarily according to ‘strain’, suggesting that the active microbiota in this region differs more between strains than between MI concentrations.

**Figure 1:**
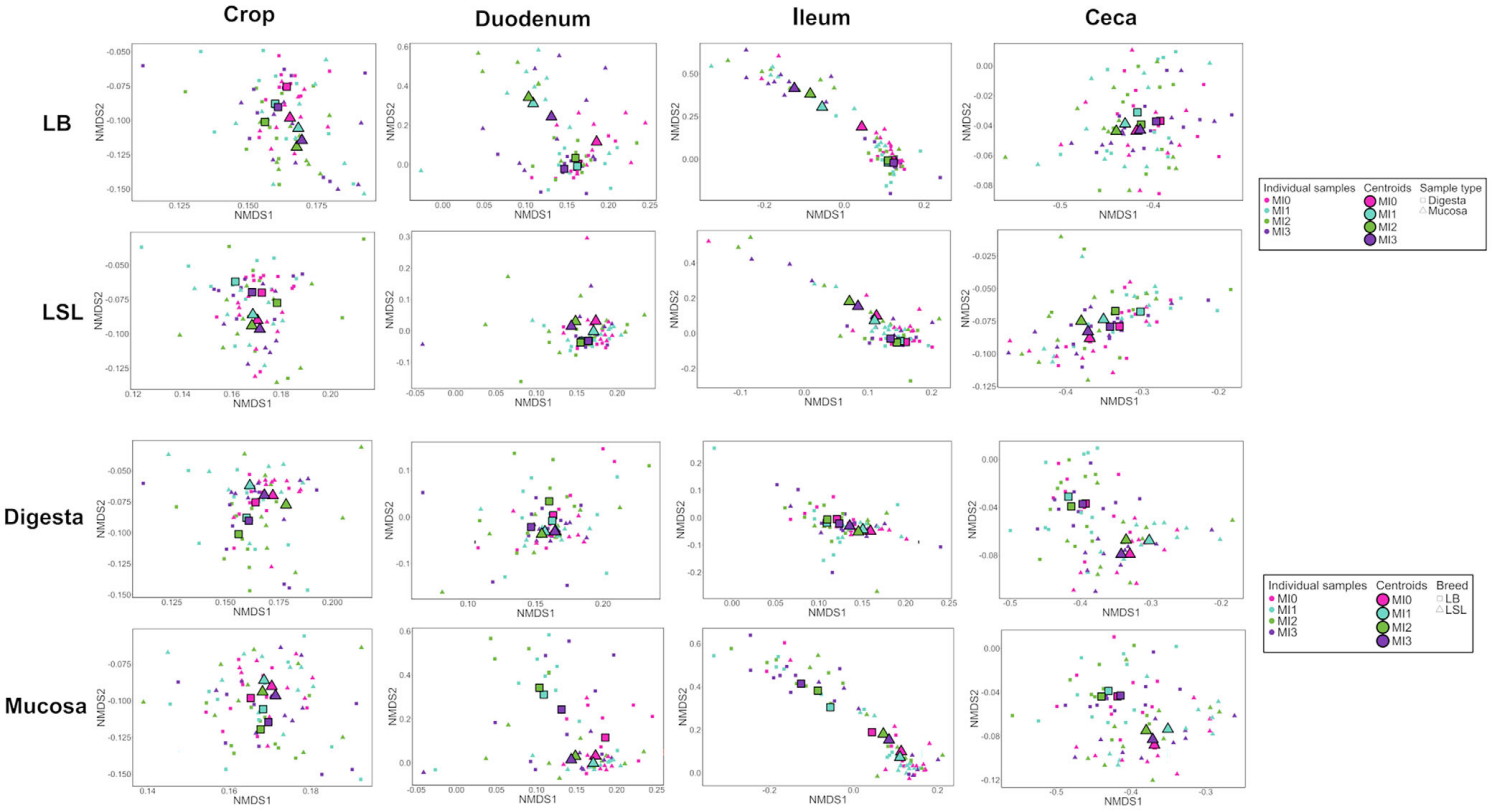
Ordination of active intestinal microbiota by GIT section, strain, sample type, and dietary MI concentration. Non‐metric multidimensional scaling (NMDS) plots based on Bray–Curtis dissimilarities at ASV level, showing the distribution of samples according to dietary treatment (MI0, MI1, MI2, MI3), sample type (digesta, mucosa), GIT section (crop, duodenum, ileum, ceca) and strain (LB, LSL). Centroids represent the multivariate mean position of each MI concentration within a given combination of strain, GIT section, and sample type.

PERMANOVA within each ‘GIT section’ and ‘strain’ combination revealed a significant ‘diet’ effect in the crop and duodenum of the LB hens, and in the ceca of both ‘strains’ (*P* < 0.05) with a trend in the ileum of LB hens (*P* < 0.1). The factor ‘sample type’ was significant in all ‘GIT sections’ (*P* < 0.05) (Supporting Information, Table S3). Across all combinations of ‘GIT section’ and ‘sample type,’ ‘diet’ showed no significant effect (*P* > 0.05), while ‘strain’ remained significant in all cases (*P* < 0.05) (Supporting Information, Table S4).

### Dietary MI induces taxon‐specific shifts without a uniform dose–response

Linear models at the genus level showed no significant differences across MI concentrations and across any GIT section (*P* > 0.05). At the ASV level, however, 66 ASVs differed significantly across MI concentrations. Taxonomically, these responders were dominated by the genera *Limosilactobacillus* (11 ASVs) and *Bacteroides* (10 ASVs) (Fig. 2; Supporting Information, Fig. S2, Tables S1 and S2).

**Figure 2:**
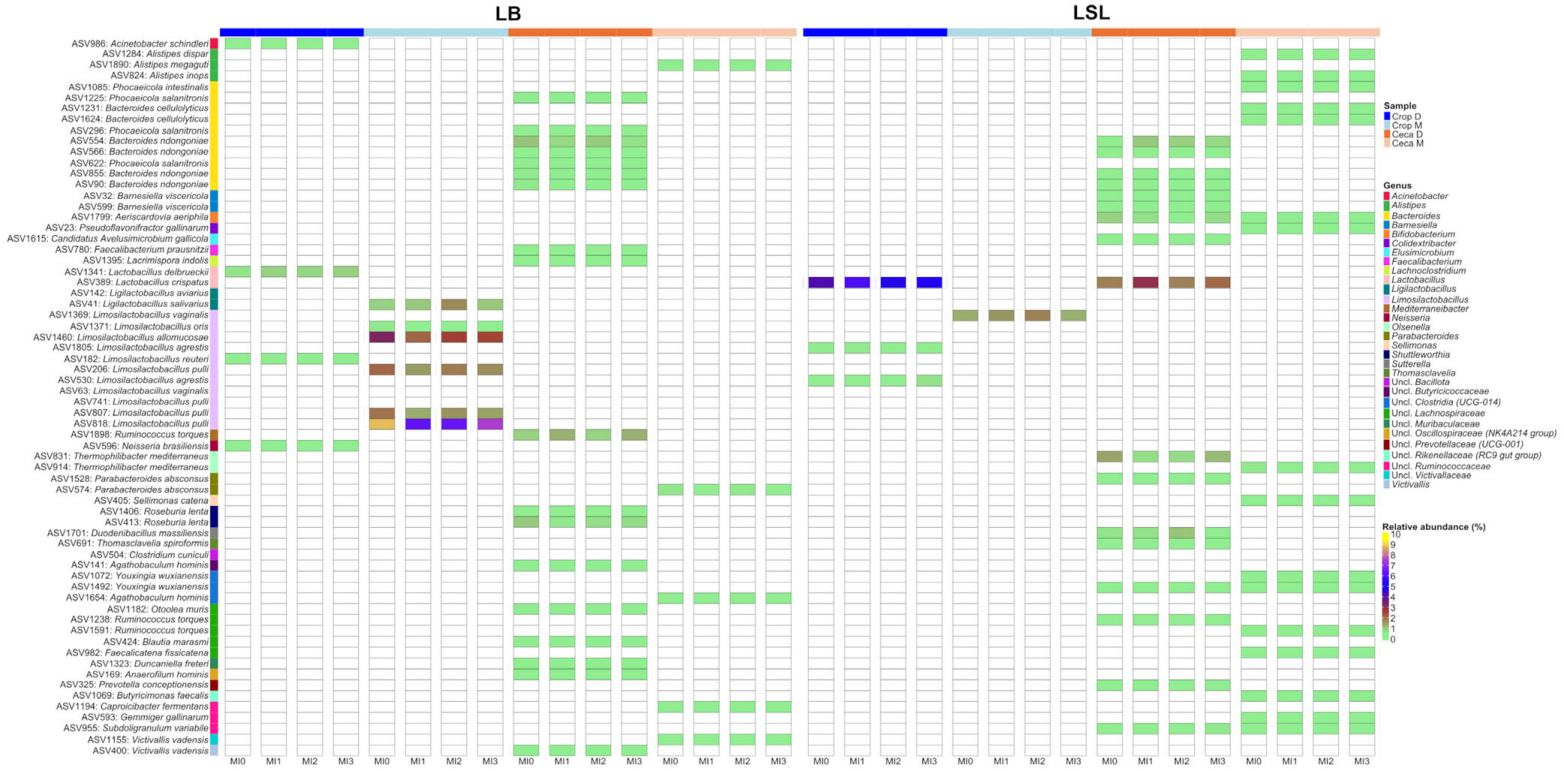
ASVs differing in relative abundance across dietary MI concentrations. Heatmap of the MI‐responsive ASVs identified by linear models at ASV level, stratified by strain (LB, LSL), GIT section (crop, ceca) and sample type (digesta = D, mucosa = M). Colors represent relative abundances, with rows corresponding to ASVs and columns to individual samples grouped by MI concentration (MI0, MI1, MI2, MI3). Only ASVs with significant effects of diet after multiple testing correction are shown.

Most of the MI‐associated differences were observed in the crop and ceca across both strains and sample types, although no consistent dose–response pattern was evident (Fig. 2). In the crop mucosa of LB strain, several *Limosilactobacillus* ASVs, including ASV818 (*Limosilactobacillus pulli*), ASV1460 (*Limosilactobacillus allomucosae*), ASV206 (*Limosilactobacillus pulli*) and ASV807 (*Limosilactobacillus pulli*) differed between MI0 and MI1, while in LSL crop digesta ASV389 (*Lactobacillus crispatus*) and ASV530 (*Limosilactobacillus agrestis*) differed significantly between MI0 and MI1 (*P* < 0.05). In LSL crop mucosa, ASV1369 (*Limosilactobacillus vaginalis*) differed between MI0 and MI2 (*P* < 0.05; Supporting Information, Table S2).

In the small intestine, LB hens showed fewer MI‐associated changes. Only one ASV was significantly affected in the ileal digesta: ASV142 (*Ligilactobacillus aviarius*) decreased under MI1 compared with MI0 and MI2 (4.0% *vs*. 4.7% and 5.7%). In contrast, LSL hens exhibited more differences in the duodenal and ileal digesta. ASV741 (*Limosilactobacillus pulli*) decreased with increasing MI dose in both regions, with significant contrasts between MI1 and MI3 in the duodenum and between MI3 and both MI0 and MI1 in the ileum (Supporting Information, Fig. S2 and Table S2).

In the ceca, several ASVs responded to MI supplementation. In LB ceca digesta, ASV1406 (*Roseburia lenta*) differed between MI0 and MI1, and additional significant differences were observed for ASV1182 (*Otoolea muris*), ASV1323 (*Duncaniella freteri*), ASV169 (*Anaerofilum hominis*), ASV413 (*Roseburia lenta*), and ASV855 (*Bacteroides ndongoniae*) between the same MI concentrations. In LSL ceca digesta, ASV855 (*Bacteroides ndongoniae*), ASV1492 (*Youxingia wuxianensis*), and ASV566 (*Bacteroides ndongoniae*) differed between MI0 and MI1. Cecal mucosa of LB hens showed fewer shifts, with only ASV574 (*Parabacteroides absconsus*) differing between MI0 and MI1. In contrast, more differences were found in the cecal mucosa of LSL hens, including ASV1799 (*Aeriscardovia aeriphila*) between MI1 and MI2, and ASV405 (*Sellimonas catena*) between MI2 and MI3 (Fig. 2; Supporting Information, Table S2). These ASVs were generally present at low relative abundances within the cecal community.

### Host genotype determines the differential abundance of key genera

The comparison of relative abundances greater than 1% at the genus level between strains within the same MI concentration, across GIT sections and sample types, showed several differences (Fig. 3, Supporting Information, Table S3). In the crop, no significant strain differences were observed (*P*_adj_ > 0.05). Significant differences in the mucosa were observed only in the duodenum for MI1, for *Lactobacillus* (LB 13.9%, LSL 54.6%) and *Limosilactobacillus* (LB 6.6%, LSL 22.7%). A few genera differed in the cecal mucosa, including *Barnesiella* in MI0 (LB 1.0%, LSL 0.4%) and *Bifidobacterium* in MI3 (LB 0.3%, LSL 0.9%). Some genera, such as *Helicobacter* in the duodenal and *Candidatus Arthromitus* in the ileal mucosa, were more abundant in the LB than in the LSL strain across different MI concentrations, but these differences were not statistically significant.

**Figure 3:**
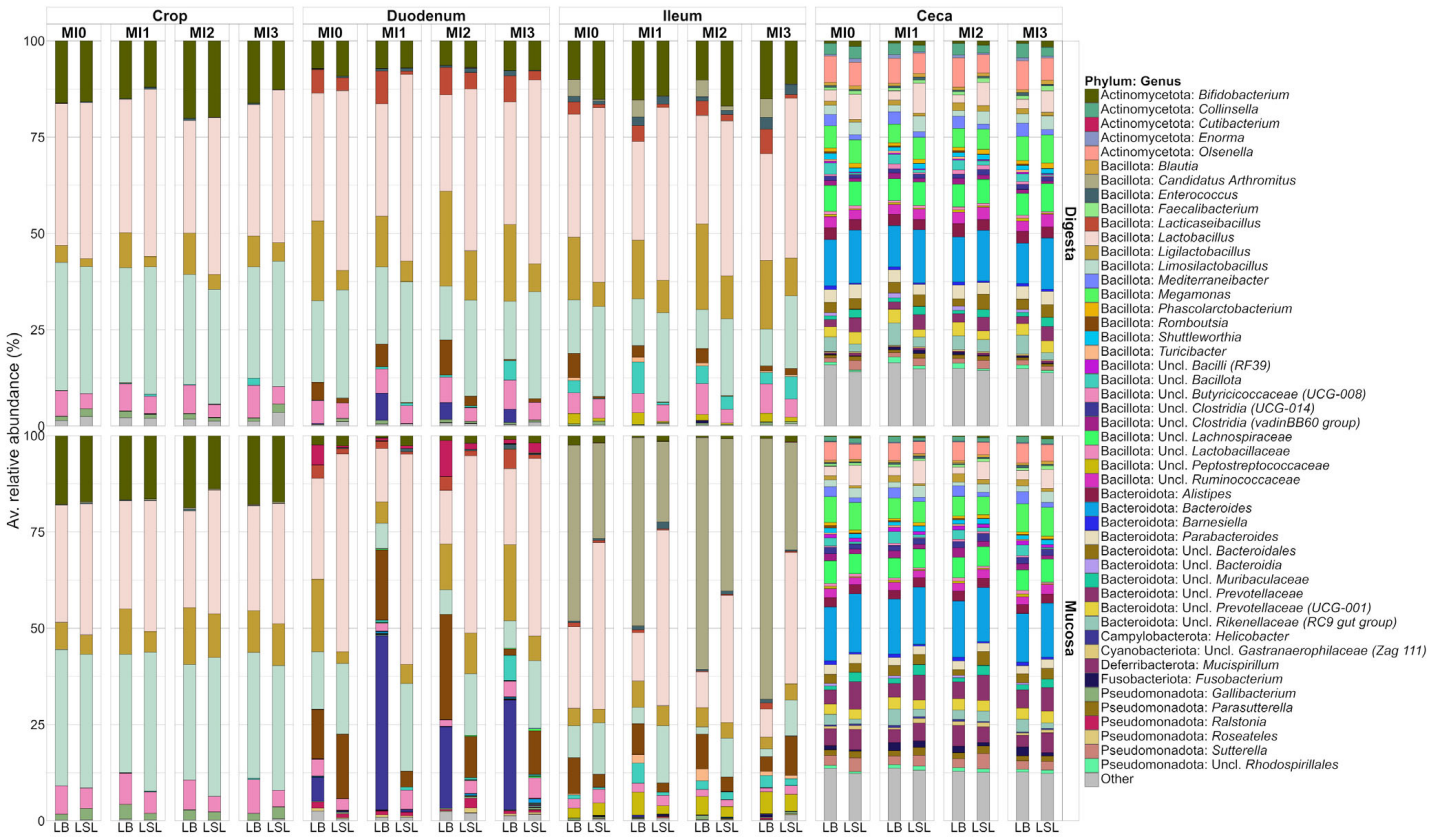
Genus‐level comparison of LB and LSL hens across GIT sections, MI concentrations and sample types. Relative abundances of bacterial genera (≥1% mean abundance) in digesta and mucosa of crop, duodenum, ileum and ceca, separated by strain (LB, LSL) and MI concentration (MI0–MI3).

In the duodenum and ileum, MI1 resulted in higher abundances of *Lacticaseibacillus* in LB digesta (8.5%) than LSL digesta (0.9%). Under the same MI level, LB hens had lower abundances of *Lactobacillus* and *Limosilactobacillus* (29.2% and 20.0%) than LSL hens (48.4% and 31.3%) in the duodenal digesta. At MI3, *Lactobacillus* remained lower in LB (31.8%) than in LSL (47.7%) duodenal digesta. In ileal digesta, higher abundances of *Romboutsia* and *Turicibacter* were observed in LB (3.0% and 1.2%) than in LSL (0.2% and 0.0%). In the ceca, several genera showed consistent strain‐related differences across MI treatments. *Lactobacillus* was more abundant in LSL digesta than in LB digesta. In contrast, *Mediterraneibacter* was enriched in LB across all MI concentrations and sample types. *Barnesiella* also showed higher relative abundance in the LB digesta, with significant differences at all MI concentrations except MI3 (Supporting Information, Table S3).

### Specific taxa correlate with growth, egg, and MI metabolism traits

Data on zootechnical traits, egg traits, and MI metabolism traits for the same hens were originally published by Sommerfeld *et al*.^3^ and used in the present study for exploratory correlation analysis. Specific taxa show weak, exploratory associations with growth, egg, and MI‐related traits. Correlation coefficients were generally low (*R*‐values mostly <0.5) and should therefore be regarded as indicative rather than evidence of causal relationships. Across GIT sections and strains, only a limited number of genera showed reproducible associations with performance‐related traits (Supporting Information, Fig. S3), egg‐related parameters (Supporting Information, Fig. S4), and MI metabolism traits (Supporting Information, Fig. S5). ADFI_Whole_ was positively associated with *Lactobacillus* in duodenal and ileal digesta but negatively associated with *Limosilactobacillus* in duodenal mucosa and *Turicibacter* in ileal mucosa. In LB hens, *Alistipes* and *Lactobacillus* in the ceca were positively related to egg weight, while in LSL hens *Ligilactobacillus* in duodenal and ileal digesta was associated with egg number and yolk MI concentration. For egg traits, correlations were more frequent in LSL than LB hens (Supporting Information, Fig. S4), but significant associations were detected in both strains and across all GIT sections and sample types. In LSL, *Ligilactobacillus* in duodenal digesta correlated positively with egg number and yolk MI concentration, and *Candidatus Arthromitus* in ileal digesta correlated positively with egg number. In the LB, *Lactobacillus* in cecal digesta and mucosa was positively correlated with egg number. MI concentrations in albumen correlated positively with *Faecalibacterium*, *Megamonas* and *Parasutterella* in the cecal digesta of LB. In LSL, MI concentration in yolk was positively associated with *Ligilactobacillus* in the ileal digesta, whereas in LB it correlated negatively with *Limosilactobacillus* in the duodenal mucosa.

Correlations with MI metabolism parameters were more frequent in LSL than LB (Supporting Information, Fig. S5). Given the modest effect sizes and the cross‐sectional nature of the data, these patterns should be considered hypothesis‐generating and require confirmation by targeted functional studies.

## DISCUSSION

### Dominant effects of GIT section and host genotype

The present study confirmed that the ‘GIT section’ was the main driver of variation in the composition of the active microbiota, with ‘strain’ as the second most important factor, in line with previous work in the same laying hen strains.^9^ This pattern is consistent with the marked morphological and physiological properties along the avian GIT,^6^ which create distinct ecological niches and support complex, section‐specific microbial communities.^7^ The concept of core microbiota has been proposed to describe key microorganisms that are stable and permanent across individuals or environments.^31^ Wang *et al*.^32^ applied this concept to identify unique and shared ASV counts among chicken breeds, and thereby revealed breed‐specific microbiota signatures. However, GIT section and sample type were not experimental treatments but repeated within‐bird biological compartments. In the overall PERMANOVA, repeated measurements were explicitly accounted for by using hen identity as the permutation stratum; the variance contributions of GIT section and sample type therefore reflect within‐bird differences rather than independent treatment effects. Strain‐related patterns were most evident in the duodenum, ileum, and ceca, particularly in digesta samples, whereas the crop showed fewer strain‐dependent differences. Sample type (digesta *vs*. mucosa) explained a smaller proportion of the total variance than GIT section and strain, but still exerted a consistent within‐bird effect on community structure, in agreement with earlier studies reporting differences between luminal and mucosa‐associated communities in chickens, which reflect the distinct microenvironment at the epithelial surface compared with the digesta.^6^, ^9^, ^33^, ^34^

The primary objective of this study was to determine whether increasing dietary MI concentrations altered the active microbiota of laying hens and whether these responses differed between LB and LSL hens. To address this question, microbial communities were evaluated separately across GIT sections and sample types because MI availability and microbial activity differ markedly along the intestinal tract. Earlier work showed that LB and LSL hens differ in their endogenous capacity to synthesize and metabolize MI, which may be related to variation in intestinal length, morphology, or absorptive capacity.^3^ These strain‐specific differences provided the rationale for stratifying the present data set by both ‘strain’ and ‘GIT section’ to characterize the active microbiota in detail. The previous observation of incomplete MI absorption in the small intestine and the indication of different absorption efficiency in the two laying hen strains further support this stratification.^3^ Specifically, the lower MI absorption indicated for LB hens in the small intestine sections implies a higher MI availability for potential MI‐utilizing bacteria in this strain.^3^ These physiological differences may also contribute to the strain‐specific microbiota profiles observed in the present study. In particular, the greater number of MI‐responsive ASVs detected in the small intestinal digesta of LSL hens, together with the stronger overall diet effects observed in several GIT sections of LB hens, suggests that host genotype modulates microbial responses to dietary MI in a compartment‐specific manner. However, the mechanisms underlying these strain‐dependent responses remain to be elucidated.

In the present study, the factor ‘diet’ had a greater influence on the total composition of the active microbiota at the ASV level in LB than in LSL, with more GIT sections showing significant MI effects in LB. The significant impact of ‘diet’ in the duodenum and the trend observed in the ileum of the LB strain are of great interest, as they point to a pronounced responsiveness of the small intestine microbiota in this strain. Rather than directly contradicting the lower MI absorption previously described for LSL^3^, these findings may indicate that the small intestinal active microbiota of LB hens has a higher functional capacity to utilize MI, thereby contributing to local MI depletion and stronger community shifts. A higher abundance or activity of MI‐consuming bacteria in the proximal intestine of LB hens would be consistent with this interpretation, but verification will require future functional analyses, such as metagenomic profiling of MI‐catabolic genes.

There is a lack of studies investigating the effect of varying MI concentrations on the intestinal microbiota of laying hens. In this context, the present work shows that MI acts as a relatively selective modulator of the active community, with most MI‐associated differences in ASV‐level relative abundances confined to the crop and ceca across both strains and sample types, and only 66 ASVs responded significantly to MI concentration. Among these, 10 and 11 ASVs belonged to the genera *Bacteroides* and *Limosilactobacillus*, respectively, indicating that MI can be effectively metabolized or indirectly influences only a subset of bacterial taxa.^2^, ^35^ Although *Lactobacillus casei* is frequently cited as prototypical MI‐utilizing species, it was not identified as MI‐responsive here, as none of the differentially abundant ASVs were assigned to this species.^2^, ^35^ A further characteristic of the MI response was the absence of a consistent dose–response pattern across the tested concentrations. None of the ASVs showed a stepwise increase or decrease in relative abundance across all four MI inclusion levels, and many shifts occurred primarily between MI0 and MI1, with higher doses failing to induce additional changes or even being associated with reduced abundances. For instance, several *Limosilactobacillus* ASVs in the crop mucosa of LB and *Bacteroides ndongoniae* in the cecal digesta of LSL hens differed mainly between MI0 and MI1, whereas ASV741 (*Limosilactobacillus pulli*) in duodenal and ileal digesta decreased at the highest MI dose. Such nonlinear responses are consistent with general models of microbial growth, where individual taxa exhibit optimal substrate concentration ranges and may experience diminishing returns or inhibition at elevated substrate levels.^36^, ^37^

Strain‐specific microbial shifts were observed within the same MI concentration. Only in the crop were no significant strain differences detected, indicating that this section harbored a relatively similar active microbiota in LB and LSL hens. In contrast, several genera showed clear strain‐related differences in the duodenum, ileum and ceca, underlining the importance of host genetic background as a determinant of intestinal microbiota composition. Together, these findings indicate that the response of the active gastrointestinal microbiota to dietary MI supplementation is not uniform across laying hen strains but is strongly influenced by host genetic background. For example, at MI1, the duodenal digesta of LB hens showed lower abundances of *Lactobacillus* and *Limosilactobacillus* than LSL hens, and *Lactobacillus* remained more abundant in the cecal digesta of LSL than LB hens across MI treatments. However, the differences in some genera, such as *Helicobacter* in the duodenal mucosa, were not statistically significant. Roth *et al*.^9^ also identified *Helicobacter* a potential pathogenic genus in intestinal samples from the same strains. In this study, its abundance increased with increasing dietary phosphorus levels. Yebra *et al*.^38^ reported that bacteria such as *Lactobacillus casei* aerobically convert MI into dihydroxyacetone phosphate, acetyl‐CoA, and CO_2_ via the iol‐catabolic pathway, highlighting a potential mechanistic link between MI availability and lactobacilli dynamics. However, these organisms metabolize MI only under aerobic conditions and are not typical members of the gut microbiota, which may explain why *Lactobacillus casei* was not detected as MI‐responsive in the present study. Instead, the observed strain‐dependent differences in *Lactobacillus* and *Limosilactobacillus* abundances, together with earlier findings that different laying hen strains adapt differently to dietary interventions, reinforce the view that host genetics critically shapes how the intestinal microbiota responds to MI supplementation.^9^

### Associations between active microbiota, performance, and MI metabolism

Intestinal microbiota has previously been associated with performance in laying hens, including body weight, egg number, and egg quality traits.^39^, ^40^, ^41^ However, to date, no studies have examined how the active intestinal microbiota relates to MI metabolism parameters in different laying hen strains. In the present study, correlations between genus‐level abundances and performance or MI‐related traits were generally weak and should therefore be regarded as exploratory.

ADFI_Whole_ was positively associated with *Lactobacillu*s in the duodenal and ileal digesta but negatively associated with *Limosilactobacillus* in the duodenal mucosa and *Turicibacter* in the ileal mucosa. Fesseha *et al*.^42^ reported that dietary supplementation with *Lactobacillus* strains (*Lactobacillus paracasei paracasei* and *Lactobacillus rhamnosus*) increased feed intake in dual‐purpose poultry, supporting a potential role of *Lactobacillus* taxa in stimulating appetite or improving feed utilization, although the modest effect sizes observed here preclude causal inference.

Egg production traits showed strain‐ and site‐specific associations with selected genera. In LSL hens, *Ligilactobacillus* in duodenal digesta was positively correlated with egg number and yolk MI concentration, whereas in LB hens *Alistipes* in cecal samples was positively associated with egg weight. These findings are consistent with previous reports that specific lactobacilli and segmented filamentous bacteria can support performance and egg quality,^43^, ^44^, ^45^ but the divergent literature on *Lactobacillus* and egg weight^9^, ^46^ indicates that microbiota–performance links are likely multifactorial and dependent on strain as well as site rather than governed by single ‘beneficial’ genera.

In both strains, MI‐related traits (yolk MI conc., albumen MI conc., phosphatase activity and ileal MI conc.) showed multiple correlations with members of the active microbiota. Among these, *Megamonas* in the cecal digesta of LB hens was positively associated with albumen MI concentration, which is consistent with reports that *Megamonas rupellensis* can utilize MI and therefore represents a plausible contributor to MI turnover in the distal gut.^35^ In LB, phosphatase activity correlated negatively with *Lactobacillus* in crop and ileal digesta, whereas in LSL hens it was positively associated with *Ligilactobacillus* in duodenal and ileal digesta, and mucosa. These strain‐specific associations contrast with the findings of Sommerfeld *et al*.,^3^ who did not detect effects of strain or diet on mucosal phosphatase activity, and therefore should be interpreted cautiously.^3^

## CONCLUSION

This study demonstrates that dietary MI supplementation induces localized and strain‐dependent alterations in the active gastrointestinal microbiota of laying hens, with potential associations with egg production traits. Although GIT section was the major source of overall microbial variation, MI supplementation affected specific bacterial taxa in a compartment‐specific manner, and these responses differed between the two commercial laying hen strains. These findings provide new evidence for interactions between dietary MI, the active gut microbiota, and host genotype, thereby extending current knowledge on the nutritional modulation of the poultry microbiome. A limitation of the present study is the exclusive use of 16S rRNA gene amplicon sequencing, which limits functional resolution and prevents direct inference of metabolic capabilities. Future research should therefore integrate metagenomics, transcriptomics, and targeted metabolite profiling to elucidate the mechanisms linking MI supplementation, microbiota shifts, and performance outcomes. In addition, longitudinal study designs could help clarify the temporal dynamics of microbial adaptation to MI and determine whether early‐life MI exposure has lasting effects on the intestinal microbiota and laying performance.

## Supporting information


**Figure S1.** Alpha diversity of the active microbiota along the GIT under graded MI supplementation. Shannon diversity indices of mucosa and digesta samples from crop, duodenum, ileum, and ceca of LB and LSL hens fed MI0–MI3 diets. Panel A shows comparisons between strains within MI concentrations and GIT sections; panel B shows comparisons between MI concentrations within each strain and GIT section.
**Figure S2.** ASVs differing in relative abundance across dietary MI concentrations. Heatmap of the MI‐responsive ASVs identified by linear models at ASV level, stratified by strain (LB, LSL), GIT section (crop, duodenum, ileum, ceca) and sample type (digesta, mucosa). Colors represent relative abundances, with rows corresponding to ASVs and columns to individual samples grouped by MI concentration (MI0, MI1, MI2, MI3). Only ASVs with significant effects of diet after multiple testing correction are shown.
**Figure S3.** Correlations between active microbiota and growth parameters. Spearman correlation network or heatmap showing significant correlations (*P* < 0.05) between genus‐level relative abundances and growth‐related traits (BW_Slaughter_, ADFI_Whole_) within each GIT section and strain. Only genera with mean relative abundance ≥1% are included.
**Figure S4.** Correlations between active microbiota and egg‐related parameters. Spearman correlation network or heatmap of significant correlations (*P* < 0.05) between genus‐level relative abundances and egg parameters (egg number, mean egg weight, egg weight oviduct, MI conc. albumen and MI conc. yolk) within each GIT section, sample type, and strain. Positive and negative correlations are indicated by color scale.
**Figure S5.** Correlations between active microbiota and MI metabolism traits. Spearman correlation network or heatmap of significant correlations (*P* < 0.05) between genus‐level relative abundances and MI metabolism parameters (phosphatase activity, ileal MI concentration) across GIT sections, sample types, and strains. Only taxa with a mean relative abundance ≥1% and significant associations after correction are shown.

**Table S1.** Output Tukey test to compare the rCLR transformed relative abundance data at the ASV level that differed.
**Table S2.** Mean relative abundances of the ASVs (%) that differed significantly between MI concentrations within the different ‘strain’, ‘GIT sections’ and ‘sample types’.
**Table S3.** Output Wilcoxon test to compare the relative abundance data at the genus level that differed significantly between the strains within the same dietary *myo*‐inositol concentration (*P* < 0.05) divided by GIT section and sample type.

## Data Availability

The data that support the findings of this study are openly available in European Nucleotide Archive (ENA) at https://www.ebi.ac.uk/ena/browser/home, reference number PRJEB121363.
